# Defining the Estimated Core Genome of Bacterial Populations Using a Bayesian Decision Model

**DOI:** 10.1371/journal.pcbi.1003788

**Published:** 2014-08-21

**Authors:** Andries J. van Tonder, Shilan Mistry, James E. Bray, Dorothea M. C. Hill, Alison J. Cody, Chris L. Farmer, Keith P. Klugman, Anne von Gottberg, Stephen D. Bentley, Julian Parkhill, Keith A. Jolley, Martin C. J. Maiden, Angela B. Brueggemann

**Affiliations:** 1 Nuffield Department of Medicine, The Peter Medawar Building for Pathogen Research, University of Oxford, Oxford, United Kingdom; 2 Mathematical Institute, University of Oxford, Oxford, United Kingdom; 3 Department of Zoology, University of Oxford, Oxford, United Kingdom; 4 Hubert Department of Global Health Epidemiology, Rollins School of Public Health, Atlanta, Georgia, United States of America; 5 Centre for Respiratory Diseases and Meningitis, National Institute for Communicable Diseases, Johannesburg, South Africa; 6 Pathogen Genomics Team, Wellcome Trust Sanger Institute, Wellcome Trust Genome Campus, Cambridge, United Kingdom; 7 Department of Medicine, University of Cambridge, Addenbrookes Hospital, Cambridge, United Kingdom; Hellas, Greece

## Abstract

The bacterial core genome is of intense interest and the volume of whole genome sequence data in the public domain available to investigate it has increased dramatically. The aim of our study was to develop a model to estimate the bacterial core genome from next-generation whole genome sequencing data and use this model to identify novel genes associated with important biological functions. Five bacterial datasets were analysed, comprising 2096 genomes in total. We developed a Bayesian decision model to estimate the number of core genes, calculated pairwise evolutionary distances (p-distances) based on nucleotide sequence diversity, and plotted the median p-distance for each core gene relative to its genome location. We designed visually-informative genome diagrams to depict areas of interest in genomes. Case studies demonstrated how the model could identify areas for further study, e.g. 25% of the core genes with higher sequence diversity in the *Campylobacter jejuni* and *Neisseria meningitidis* genomes encoded hypothetical proteins. The core gene with the highest p-distance value in *C. jejuni* was annotated in the reference genome as a putative hydrolase, but further work revealed that it shared sequence homology with beta-lactamase/metallo-beta-lactamases (enzymes that provide resistance to a range of broad-spectrum antibiotics) and thioredoxin reductase genes (which reduce oxidative stress and are essential for DNA replication) in other *C. jejuni* genomes. Our Bayesian model of estimating the core genome is principled, easy to use and can be applied to large genome datasets. This study also highlighted the lack of knowledge currently available for many core genes in bacterial genomes of significant global public health importance.

## Author Summary

Whole genome sequencing has revolutionised the study of pathogenic microorganisms. It has also become so affordable that hundreds of samples can reasonably be sequenced in an individual project, creating a wealth of data. Estimating the bacterial core genome – traditionally defined as those genes present in all genomes – is an important initial step in population genomics analyses. We developed a simple statistical model to estimate the number of core genes in a bacterial genome dataset, calculated pairwise evolutionary distances (p-distances) based on differences among nucleotide sequences, and plotted the median p-distance for each core gene relative to its genome location. Low p-distance values indicate highly-conserved genes; high values suggest genes under selection and/or undergoing recombination. The genome diagrams depict areas of interest in genomes that can be explored in further detail. Using our method, we analysed five bacterial species comprising a total of 2096 genomes. This revealed new information related to antibiotic resistance and virulence for two bacterial species and demonstrated that the function of many core genes in bacteria is still unknown. Our model provides a highly-accessible, publicly-available tool to use on the vast quantities of genome sequence data now available.

This is a *PLOS Computational Biology* Methods *article.*

## Introduction

The advent of next-generation sequencing (NGS) has greatly increased the number of bacterial genomes sequenced and made available for study in public databases such as GenBank, the Sequence Read Archive and European Nucleotide Archive (ENA) [1]–[3]. Increasing computational power allows for comparative genomics studies involving hundreds or even thousands of sequences, but large scale computational resources are not available to all researchers. Developing methods for analysing large datasets that capitalise on the computational power of modern desktop computers will make comparative genomics analyses much more accessible to the wider research community, allowing this vast quantity of data to be analysed more extensively.

A bacterial species can be defined by its pan-genome, which consists of a core genome conventionally defined as those genes present in all isolates, and an accessory genome, which includes the genes absent from one or more isolates or unique to a given isolate (note that we use the term “gene” here to refer to a putative protein-coding sequence) [4]. Identifying the core complement of genes in a bacterial species is often the first step in population genomics studies and the core genome can be defined in different ways. The most conservative and most frequently employed method is to only include genes present in 100% of isolates within the study population; however, this presents problems related to both biological sampling and the sequencing technology. Any collection of isolates is a subset of the entire population for the species of interest, and if the subset of isolates has limited genetic diversity then the number of “core” genes shared by all isolates in that sample will be higher than in a dataset which is genetically more diverse. This is not necessarily a problem, unless the intention is to extrapolate the findings to the wider bacterial population. Another biological limitation to using a 100% cut-off for inclusion in the core genome is that there may be rare variant strains which are missing genes that would otherwise be considered core genes. These variant strains may survive long enough to be sampled, potentially skewing the analyses. More generally, the size of the core genome is dependent on the size of the data set, with the core genome decreasing in size as more genomes are added to the analysis [4].

A large proportion of the bacterial genome sequences available at the time of writing are produced using next-generation sequencing platforms such as Illumina or Roche 454, so that even high-quality assemblies remain as incomplete or “draft” genomes. This is acceptable for most studies, but analyses of these genomes may exclude a gene from a list of core genes simply because it contains a sequence gap or is otherwise incomplete at that locus in the assembly of one or a few genomes. This assumes that the sequences being compared are all full-length: if an analysis accepts less than full-length coding sequences then gaps may not be an issue, but there will be other challenges with using incomplete sequence data, e.g. calculating pairwise distance (p-distance) measures.

If a definition of core genes as those found complete in all isolates in the dataset is too conservative, then the problem becomes that of determining an acceptable limit to the number of isolates missing any particular gene. One approach is to plot a frequency distribution that indicates how many genes are present in all isolates, or are missing in one or more isolates within the study population (Figure S1). For some bacterial species, there is a reasonably clear delineation between genes present in a large proportion of the study population versus those that are infrequent or rare, but for other bacterial species it is not clear.

Rather than make an arbitrary decision, we developed a statistical model for estimating the core genome that can be applied to different bacterial species by formalising the decision in the language of probability. The aim was to develop a Bayesian decision model to identify the genes found in what we will call the “estimated core genome” and apply this decision model to several large bacterial genome datasets. We described the nucleotide sequence diversity for each gene in the estimated core genome and considered how core genome sequence diversity varied across unrelated bacterial species. Finally, we depicted the data in a way that allowed us to explore the sequence data in greater detail and generate testable hypotheses about the estimated core genome.

The five bacterial species chosen for inclusion in this study were disease-causing organisms responsible for a large proportion of the global bacterial disease burden: *Streptococcus pneumoniae* (respiratory disease, the most important cause of infectious disease mortality); *Campylobacter jejuni* (gastrointestinal disease); *Neisseria meningitidis* (meningitis); *Staphylococcus aureus* (skin and soft tissue infections); and *Helicobacter pylori* (gastrointestinal ulcers).

## Results

### Description of the datasets used in analyses

**Figure 1:**
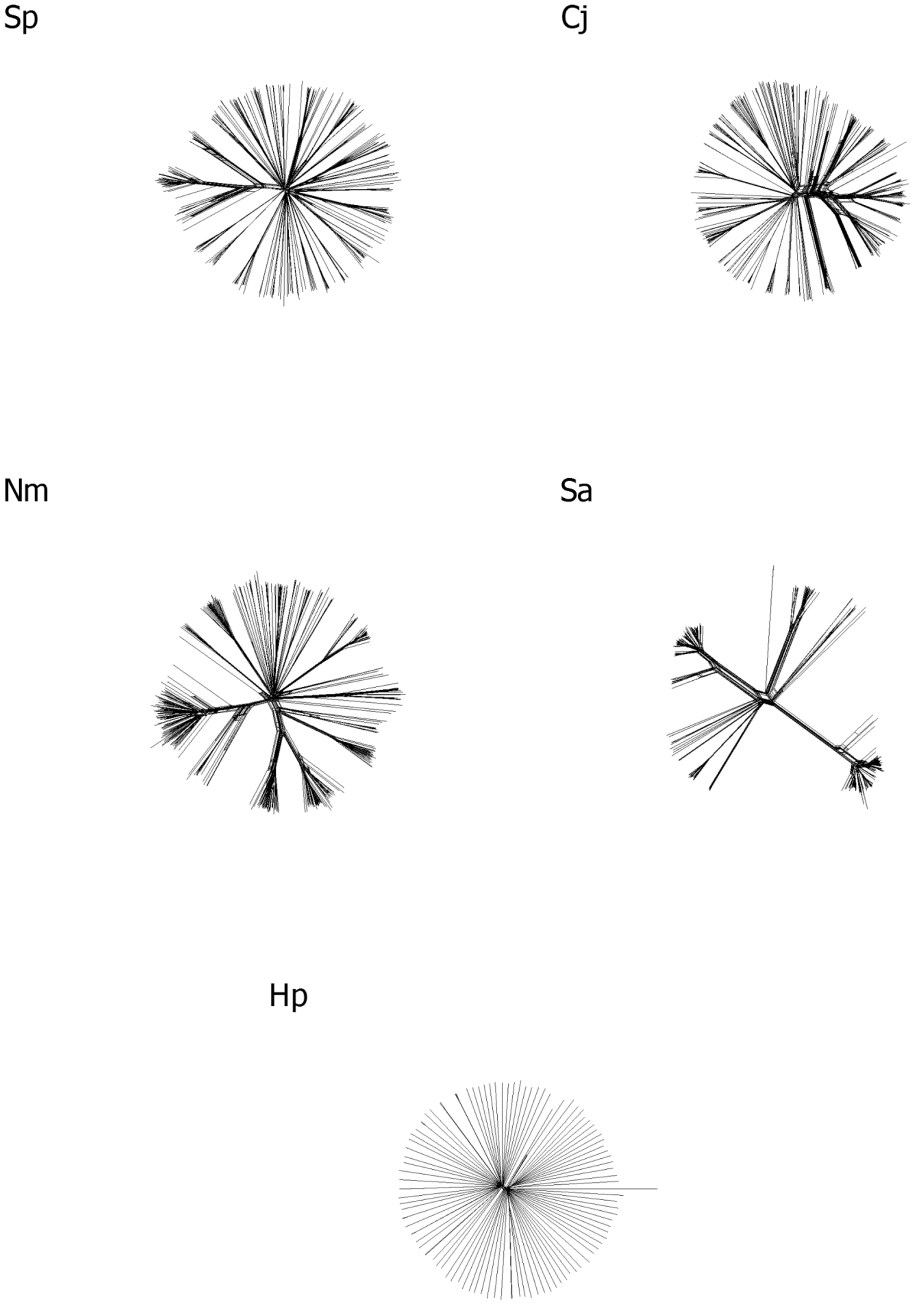
Neighbor-Net phylogenetic networks for the five species included in this study. Neighbor-Net diagrams for each species as follows: Sp) *S. pneumoniae*; Cj) *C. jejuni*; Nm) *N. meningitidis*; Sa) *S. aureus*; and Hp) *H. pylori*. Scale bars for each individual diagram were too small to be visualised and thus were removed for illustrative purposes.

In total, 2096 genomes were analysed across the 5 different bacterial species (Table 1 and Datasets S1). A phylogenetic network for each dataset was derived using Neighbor-Net [5] as part of the initial Genome Comparator [6] analyses (see Methods for a description of the Genome Comparator program); these diagrams demonstrated the overall diversity of the genomes in each study dataset (Figure 1).

**Table 1:** Summary of study datasets.

Bacterial species	Study dataset	No. of genomes	Years of isolation	No. of countries represented	No. of STs^a^	No. of CCs^a^
*S. pneumoniae*	Global Historical Collection	336	1916–2008	32	163	74
*C. jejuni*	Oxfordshire Human Surveillance 2011	601	2011	1 (UK)	134	29
*N. meningitidis*	MRF Meningococcus Genome Library	518	2010–2011^b^	3^c^	198	24
*S. aureus*	rMLST BIGSdb database^d^	534	1955–2011	27^e^	25	11
*H. pylori*	rMLST BIGSdb database	107	–f	10	–f	–f

a STs = sequence types; CCs = clonal complexes.

b Four historical isolates from 1976, 1983 (n = 2) and 1986 were also included.

c One historical isolate was from The Gambia and another was from Norway. All other isolates were from the UK.

d http://pubmlst.org/rmlst/.

e 39% (n = 210) of the genomes were from the UK; 18% (n = 94) genomes were from unknown locations.

f The year of isolation for nearly all of the genomes was unknown; STs and CCs for *H. pylori* were not defined (see main text).

The *S. pneumoniae* (pneumococcal) dataset consisted of 336 genomes for isolates of 39 different serotypes collected over 90 years (1916–2008) from at least 32 countries around the world. The isolates were recovered from individuals of a wide range of ages, including isolates from patients with disease and isolates recovered from healthy individuals. The multilocus sequence typing (MLST) data revealed 163 sequence types (STs), which could be clustered into 74 different clonal complexes (CCs) indicative of isolates with shared ancestry (Tables 1 and S1).

The largest genome dataset analysed was that of *C. jejuni* (N = 601 genomes). Isolates were recovered from human stool samples collected from patients in Oxfordshire, United Kingdom (UK) with gastroenteritis during 2011. 134 STs from 29 CCs were characterised in this collection, which was representative of the broader *C. jejuni* population genetic diversity [7], [8].

The *N. meningitidis* (meningococcal) dataset was comprised of 518 genomes and these isolates were collected nearly exclusively from patients residing in England, Wales and Northern Ireland in the 2010/11 epidemiological year, apart from 4 historical isolates from Norway (1976), The Gambia and UK (1983) and UK (1986). The 2010/11 genomes are part of the Meningitis Research Foundation Meningococcus Genome Library, which contains genomes from all culture-confirmed cases of meningococcal disease submitted to the Meningococcal Reference Unit in 2010/11 and 2011/12. Isolates of seven serogroups were included, mostly serogroup B (n = 394), Y (n = 74) and W-135 (n = 27). 198 STs were represented by the isolates and the STs clustered into 24 CCs. Culture-confirmed cases of meningococcal disease are largely representative of the England and Wales disease-causing *N. meningitidis* population as described previously [9].

The *S. aureus* dataset was large (N = 534 genomes) but genetically less diverse (25 STs and 11 CCs; Tables 1 and S1) than other datasets, since the analyses were restricted to methicillin-resistant *S. aureus* (MRSA) only. Most of the publicly-available genomes that are already published are of a limited number of CCs, predominantly the MRSA CCs that are epidemiologically the most important. The MRSA isolates were recovered from patients in 27 countries, although 39% of isolates were recovered in the UK. The *H. pylori* dataset included 107 genomes and 82% of the collection was from the USA, Canada or Japan. Only limited additional metadata were available for these isolates.

### Estimated core genomes for each bacterial species dataset

The size of the reference genomes used in the Genome Comparator analyses for each dataset varied from 1.6 to 2.8 Mb, and the total number of genes in each reference genome ranged from 1566 to 2547 (Table 2). There were small numbers of unique loci, i.e. genes found only in the reference genome and/or present in only one genome: *S. pneumoniae* (n = 6); *C. jejuni* (n = 6); *N. meningitidis* (n = 7); *S. aureus* (n = 4); and *H. pylori* (45).

**Table 2:** Summary of estimated core genome analyses.

Bacterial species	Reference genome	Reference genome size (Mb)	Reference genes (n)	% of genomes that possess each core gene	Estimated core genes (n)
*S. pneumoniae*	ATCC700669	2.22	1990	≥99.7	851
*C. jejuni*	NCTC11168	1.64	1623	≥99.8	866
*N. meningitidis*	FAM18	2.19	1917	≥99.8	744
*S. aureus*	HO5096_0412	2.83	2547	≥99.8	242
*H. pylori*	26695	1.67	1566	≥99.1	244

An initial BLASTN criteria of 70% identity was chosen, which allowed for the identification of variable sequences among conserved gene classes [10] and avoided bias towards reference-specific sequences, and 100% sequence alignment, which means that coding sequences occurring at the ends of contigs or with gaps were therefore not included. Lowering the BLASTN criteria to 70% identity and 90% alignment increases the number of genes in the estimated core, as partial gene sequences will be detected and included (Table S2), which may be more suitable for other user-specific analyses but is not ideal for the calculation of p-distances to estimate sequence diversity.

The smallest estimated core genomes were those of MRSA and *H. pylori* (242 and 244 genes, respectively; Table 2) and the *S. pneumoniae*, *C. jejuni* and *N. meningitidis* core genomes were similar in size, ranging from 744 to 866 genes. The percentage of genomes within a dataset that possessed each estimated core gene ranged from ≥99.1% to ≥99.8%. The number of putative paralogues identified in the initial Genome Comparator analyses varied from 1 in *C. jejuni* to 40 among the *S. pneumoniae* genomes, and these genes were removed from further analyses (lists of putative paralogues for each species are provided in Table S3). If putative paralogues had not been removed, one would have been included in each of the estimated core genomes of *S. pneumoniae* and *N. meningitidis*.

### Calculated pairwise evolutionary distance values (p-distances)

**Figure 2:**
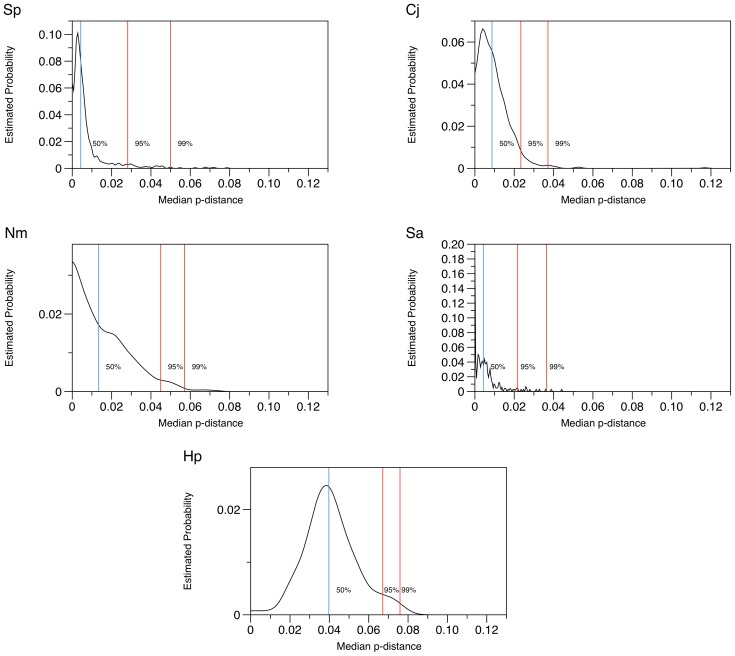
Estimated probability densities of median p-distances. The estimated probability density function was plotted for each bacterial species as follows: Sp) *S. pneumoniae*; Cj) *C. jejuni*; Nm) *N. meningitidis*; Sa) *S. aureus*; and Hp) *H. pylori*.

Within each genome dataset, median p-distance values were calculated for each of the estimated core genes and the estimated probability density function was plotted for each bacterial species (Figure 2). The estimated probability density function plotted a smoothed histogram of the median p-distances vs. the estimated probability (relative frequency) of each p-distance value. The shape of the graphs for *S. pneumoniae* and *C. jejuni* were similar, showing a large peak of very small p-distance values, i.e. highly conserved genes, but these were entirely different from the graphs depicting the data for the other bacterial species. Each graph is an indication of the overall sequence diversity of the set of estimated core genes for that particular genome dataset.

**Figure 3:**
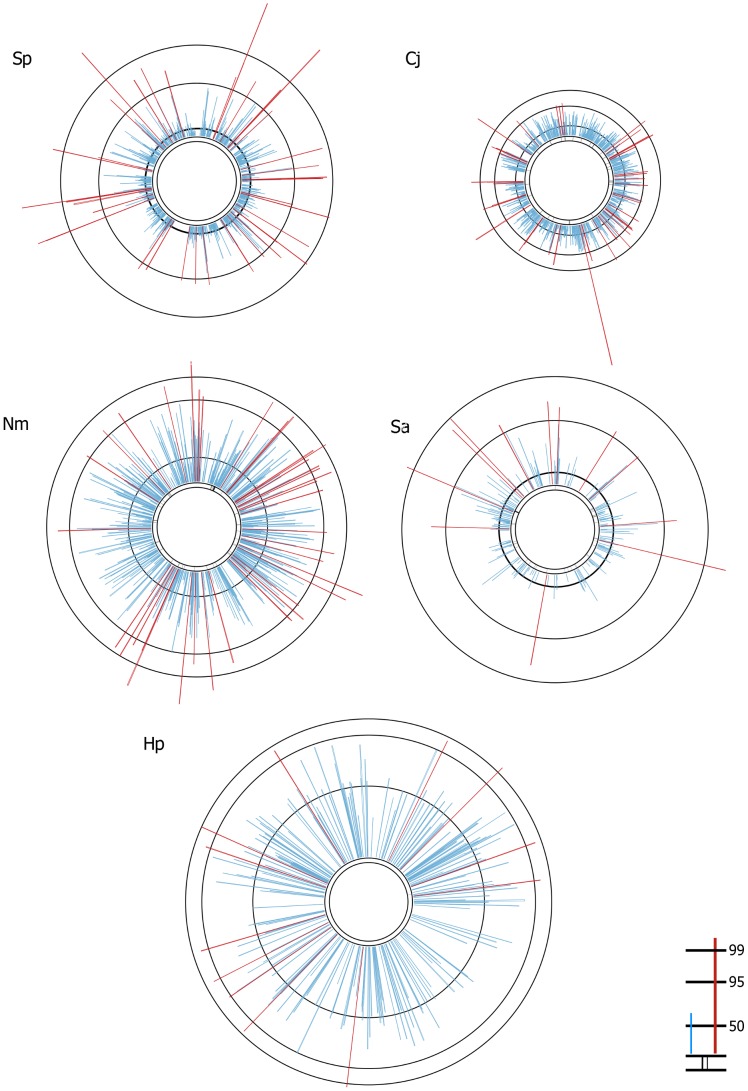
Distribution of core genes and median pairwise distances. Median p-distance plotted against genome position, relative to the reference genome, for Sp) *S. pneumoniae* (ATCC700669, 2.2 Mb); Cj) *C. jejuni* (NCTC11168, 1.64 Mb); Nm) *N. meningitidis* (FAM18, 2.19 Mb); Sa) *S. aureus* (HO5096_0412, 2.83 Mb); and Hp) *H. pylori* (26695, 1.67 Mb). The length of each line on a genome diagram was scaled according to the maximum p-distance value calculated for that particular bacterial species. The three outer rings mark the 99^th^, 95^th^ and 50^th^ percentiles. Median p-distances above the 95th percentile were coloured red; median p-distances above zero but less than the 95th percentile were coloured blue. The innermost pair of rings marks the position of genes with a median p-distance of zero.

The median p-distance value for each estimated core gene was then plotted against its position in the reference genome and illustrated as a circular bacterial chromosome (Figure 3). The length of each line indicates the median p-distance value for that gene. The estimated core genes were distributed around each genome and accessory regions in the reference genomes (e.g. ICE elements or phage genes), were observed as gaps where no core genes clustered. Estimated core genes with p-distances above 0 but less than the 95^th^ percentile (blue lines) and those above the 95^th^ percentile (red lines) stood out in a pattern on each genome diagram and allowed for an evaluation of specific genes and gene clusters in the genome, as demonstrated below. Table S4 lists all the estimated core genes and p-distances for each bacterial species. All genes with a p-distance value greater than the 95^th^ percentile for each bacterial species, and the Cluster of Orthologous Groups (COG) functional category for each of those genes, are listed in Table S5.

### Three case studies that expanded the initial analyses of the estimated core genome

#### Case study 1: Investigate the core genes in *C. jejuni* and *N. meningitidis* with p-distances above the 95^th^ percentile

Forty-three estimated core genes in *C. jejuni* were over the 95^th^ percentile (Figure 3-Cj). 26% (n = 11; range of p-distances 0.026–0.118) of these genes were hypothetical proteins or of unknown function, and 19% (n = 8) were associated with coenzyme metabolism (Tables 3 and S5). The *C. jejuni* genome diagram also clearly identified a gene in the estimated core that had a much higher p-distance value (0.118) than the rest of the core genome (the longest red line in Figure 3-Cj). Cj0809c was annotated in the reference genome as a putative hydrolase. A Pfam [11] search of the Cj0809c amino acid sequence returned a match to the Lactamase_B superfamily and a BLAST search against the NCBI non-redundant database found orthologues of the gene annotated as metallo-beta-lactamase in other *C. jejuni*. A BLAST search of the KEGG database [12] resulted in hits to sequences annotated as beta-lactamase, metallo-beta-lactamase and thioredoxin reductase (NADPH) genes in other *C. jejuni* genomes.

**Table 3:** Cluster of Orthologous Groups (COG) functional groups for the estimated core genes with p-distances greater than the 95^th^ percentile in the *C. jejuni* and *N. meningitidis* genomes.

		*C. jejuni*		*N. meningitidis*	
COG functional group		no.	%	no.	%
R/S	hypothetical protein^a^	11	25.6	9	24.3
H	coenzyme metabolism	8	18.6	7	18.9
J	translation	1	2.3	5	13.5
M	cell wall membrane envelope biogenesis	4	9.3	4	10.8
E	amino acid metabolism and transport	2	4.7	4	10.8
L	replication and repair	0	0.0	3	8.1
C	energy production and conversion	4	9.3	2	5.4
T	signal transduction	1	2.3	1	2.7
F	nucleotide metabolism and transport	1	2.3	1	2.7
P	inorganic ion transport and metabolism	0	0.0	1	2.7
G	carbohydrate metabolism and transport	3	7.0	0	0.0
O	post-translational modification, protein turnover, chaperone	3	7.0	0	0.0
I	lipid metabolism	2	4.7	0	0.0
D	cell cycle control and mitosis	1	2.3	0	0.0
K	transcription	1	2.3	0	0.0
N	cell motility	1	2.3	0	0.0
**Total**		**43**	**100**	**37**	**100**

a Includes genes in COG categories R (general functional prediction only) and S (function unknown), and genes otherwise annotated as hypothetical proteins.

Beta-lactamases and metallo-beta-lactamases are enzymes that provide resistance to a wide range of broad-spectrum beta-lactam antibiotics. Thioredoxin reductase is part of the thiol redox system, which reduces oxidative stress and is essential for DNA replication; it has also recently been proposed as a possible novel antimicrobial target site [13], [14]. The high level of sequence diversity observed in Cj0809c means that it is likely under selective pressure and/or is a site of frequent recombination, and further investigation of the true function of Cj0809c as well as the different sequence variants of this particular gene is necessary.

Overall, 37 estimated core genes in *N. meningitidis* had median p-distance values over the 95^th^ percentile threshold and these were categorised into 12 different COG functional categories (Tables 3 and S5). Similar to the *C. jejuni* data, 24% (n = 9) encoded unknown hypothetical proteins or genes with only a general predicted function and their calculated p-distances ranged from 0.045–0.073. The two core genes with the highest p-distance values (NMC1154 and NMC0661) also encode putative proteins with unknown functions. NMC1154 is adjacent to *ribA*, which encodes a GTP cyclohydrolase II (*ribA* has been proposed as a novel antimicrobial target site [15]) and NMC0661 is a hypothetical protein, possibly in the YicC-like family of bacterial proteins, which are poorly characterised but may play a role in stationary phase survival [16].

There were also several clusters of red lines in the *N. meningitidis* genome, indicating regions of the genome with higher sequence diversity among the estimated core genes (Figure 3-Nm). One gene cluster encodes proteins in the NADH dehydrogenase complex (p-distances for each core gene in this region range from 0.021–0.055; Tables S4 and S5). NADH dehydrogenase is involved in oxidative phosphorylation and has recently been identified as a region of the *N. meningitidis* genome that has undergone intragenic recombination; authors have speculated that sequence differences in this gene region may be related to differences in fitness and possibly virulence [17], [18].

**Figure 4:**
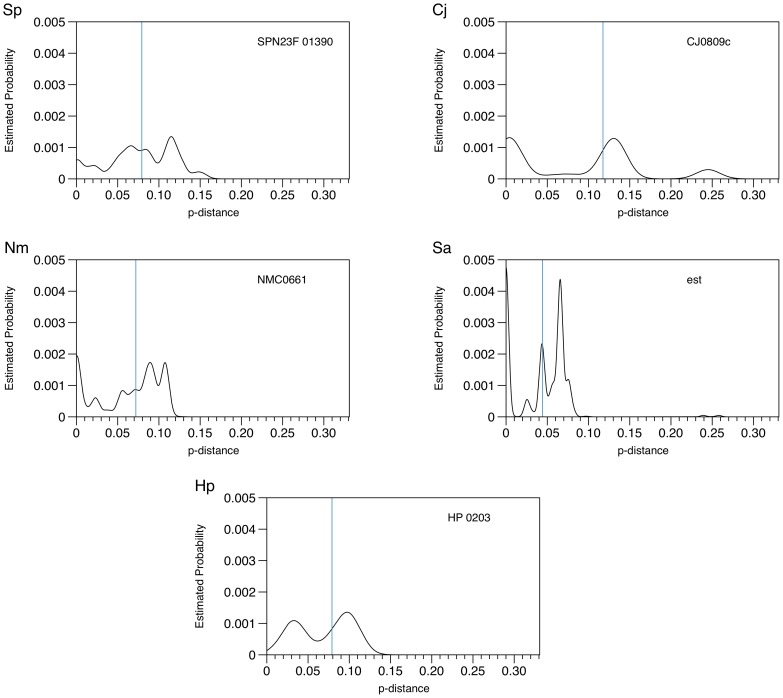
Estimated probability density of p-distances for the core gene with the highest median p-distance. Probability distributions of p-distances for the core gene with the highest median p-distance for: Sp) *S. pneumoniae*; Cj) *C. jejuni*; Nm) *N. meningitidis*; Sa) *S. aureus*; and Hp) *H. pylori*. Gene names are given in the corner of each graph.

We were also interested in exploring the matrix of calculated p-distances across all nucleotide sequences for any particular core gene, which were summarised as a median p-distance and plotted on the genome diagram. This provided insight into the range of calculated p-distances for a core gene and an indication of whether there was any particular clustering of p-distances within that gene. To investigate this, the estimated probability density graph for the core gene with the highest median p-distance value for each of the bacterial species was generated (Figure 4).

The graphs varied widely in shape and height, and the graphs for Cj0809c and HP0203 suggested that there were clusters of sequences that were likely to be biologically informative. Overall, these graphs suggest that the median p-distance value is a suitable summary statistic to represent the sequence diversity of a particular core gene across all genomes analysed, and as such it provides a useful starting point for exploring a very large set of core genes; however, a gene-specific plot of the range of p-distances should also be considered as it may reveal informative biological patterns in the data. Statistical analyses of the pairwise p-distance matrix would be required for further inference to be made.

#### Case study 2: A comparison of Bayesian vs. COGs methodologies to estimate the core genome of *S. pneumoniae*

**Figure 5:**
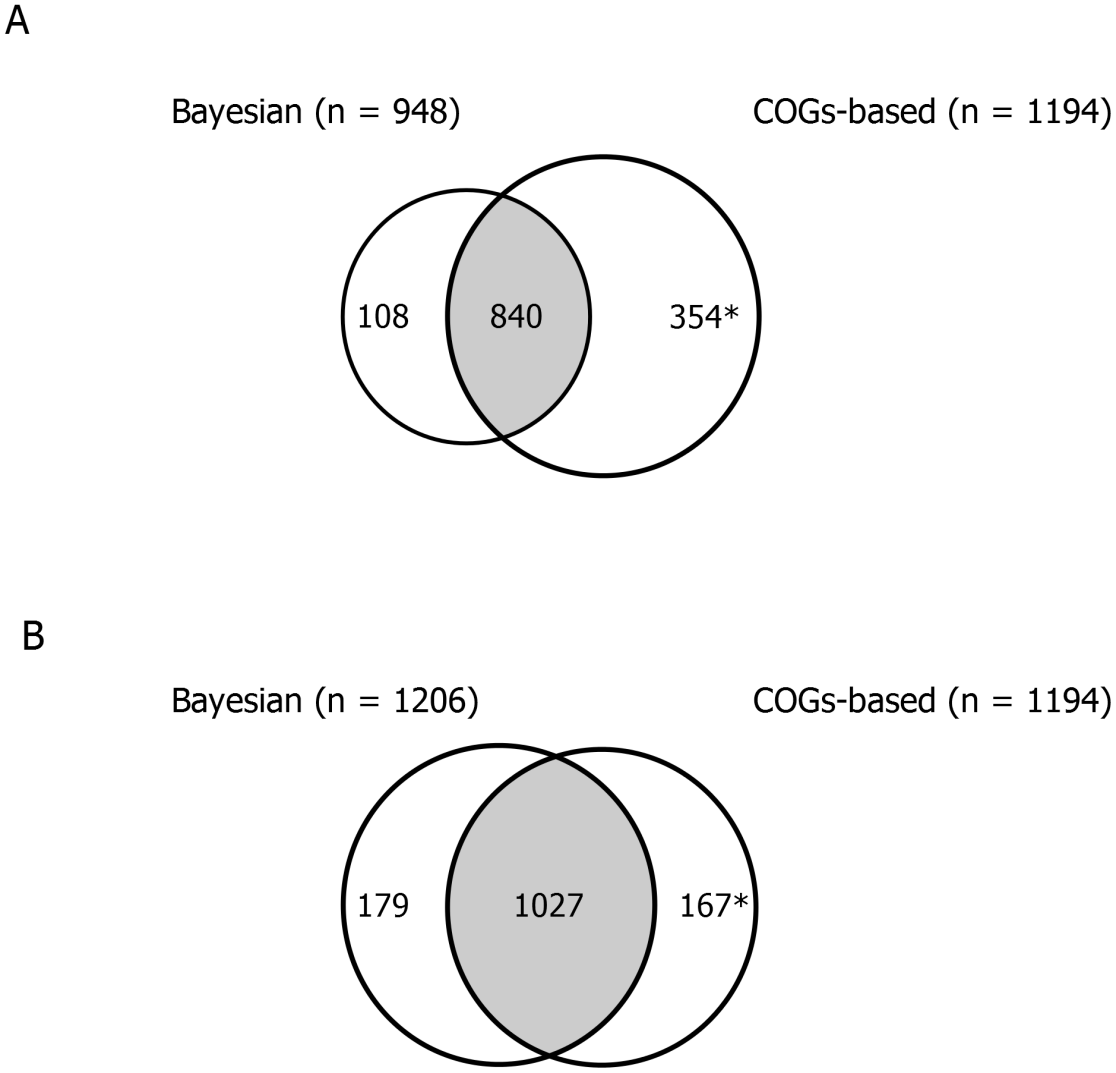
A comparison of the results of two different methodologies for estimating the core genome, using the 616 *S. pneumoniae* genomes from Massachusetts [19] as the assessment dataset. Comparison of the number of core genes identified using the Bayesian method versus the number of core genes identified using the COGs-based method. A) Results obtained using an initial BLAST cut-off of 70% identity and 100% sequence alignment for the Bayesian analysis; and B) Results obtained using an initial BLAST cut-off of 70% identity and 90% sequence alignment for the Bayesian analysis. *Note that the total includes 10 predicted coding regions that are not in the ATCC 700669 reference genome.

We were interested in comparing our Bayesian model to that of a recently published study that estimated the core genome among *S. pneumoniae* using a COGs-based methodology [19]. Croucher and colleagues generated whole genome sequence data for 616 pneumococcal carriage isolates collected during three discrete time periods between 2001–2007 from healthy children <5 years of age in Boston, Massachusetts. Putative protein-coding sequences from all genome assemblies were extracted and grouped into COGs; COGs present in 100% of genomes in a single copy were defined as core genes. Overall, the COGs-based method estimated the size of the core genome in the Massachusetts *S. pneumoniae* dataset to include 1194 genes, whereas our Bayesian analysis of the same dataset estimated 948 core genes. 840 genes were common to both estimations and 108 and 354 genes were unique to each methodology (Figure 5A; Table S6).

An explanation for the difference in core genome estimates was that we set the Genome Comparator analysis to only consider full-length genes (100% alignment of the putative coding sequence), whereas partial sequences were included in the COGs-based methodology. To test this, we reran the Genome Comparator analysis and lowered the threshold to 90% sequence alignment (i.e. up to 10% of the sequence could be missing and the gene would still be included in the estimated core genome). The result was that the sets of estimated core genes were very similar: 1206 vs. 1194 genes, of which 1027 genes were found in both lists (Figure 5B).

It is useful to note here that the output generated using our methodology includes a list of the estimated core genes (named according to the reference genome), their predicted functions (including hypothetical proteins) and location in the genome (relative to the reference genome). The COGs-based analysis outputs data for COG groups, which requires the user to undertake additional processing steps to acquire the corresponding list of gene names, sequences and genome position.

#### Case study 3: Comparison of the estimated core genome of specific MRSA CCs and STs

The estimated core genome for the MRSA dataset derived from the Bayesian model was much smaller than previous estimates of the number of core genes in *S. aureus*/MRSA. Prior estimates of the *S. aureus* core genome varied from 1492 to 2266 genes [20]–[22], whilst the figure we obtained was only 242 genes. This was due to a number of factors: i) recently published analyses of larger MRSA genome datasets were restricted to a single CC, in which case the set of genes in the estimated core will be larger since the genomes share common ancestry [20], [23], [24]; ii) the much larger sample size of our dataset (534 genomes) in comparison to previous estimates using datasets of 14–63 MRSA genomes [20]–[22]; and iii) the exclusion of partial gene sequences (Table S2) which means we have a very conservative estimate of the genes found in the MRSA core genome. To test whether our estimate of a smaller MRSA core genome may also be associated with major core genome differences between unrelated CCs, we reanalysed the MRSA genome data in Genome Comparator by running the two largest CCs, 5 and 22, individually. The CC-specific data were then compared with respect to the number of estimated core genes and the COG functional groups associated with each unique set of core genes.

CC5 and CC22 were comprised of 207 and 235 genomes, respectively, in our MRSA dataset. 81% (167/207) of genomes in CC5 were ST239, a widely-distributed, multidrug-resistant clone that is among the most common MRSA lineages worldwide [25], [26]. The predominant genotype in CC22 was ST22 (186/235; 79%), also known as EMRSA-15 and responsible for 80% of healthcare-associated MRSA infection in the UK [27].

**Figure 6:**
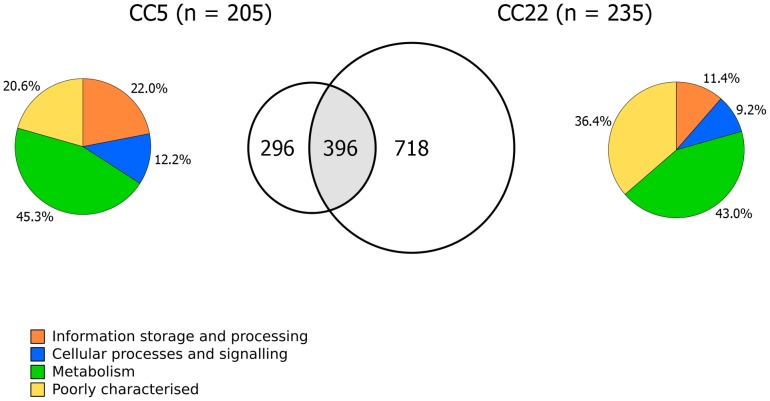
Comparison of *S. aureus* clonal complexes CC5 and CC22. Venn diagram of the comparison between the estimated core genes for CC5 and CC22, along with pie charts showing the COG functional categories associated with the genes that were unique to each dataset.

Core genomes of 692 and 1114 genes were estimated for CC5 and CC22, respectively, and 396 genes were common to both CCs (Figure 6; Table S7). A total of 296 genes were defined as core genes in CC5 but not in CC22: 45% of these were associated with metabolic functions, 22% with information storage/processing, 12% with cellular processes and signalling, and 21% were poorly categorised or with unknown function. In contrast, 718 estimated core genes in CC22 were not part of the core genome of CC5: the proportions of unique genes associated with metabolism or cellular processes/signalling were similar to that of CC5, but half as many were associated with information storage/processing and notably, over one-third (n = 261) of unique core genes in CC22 were poorly categorised or with unknown function. Given that CC22 is a major healthcare-associated MRSA clone, an argument could be made for identifying and discerning the functions of these unique core genes.

## Discussion

This study exploited the large volume of publicly-available whole genome sequence data to outline a method for analysing bacterial genomes in a straightforward way, using web-based tools and computer programmes that run on modestly-powered computers. The analyses described here do not require access to supercomputers. The resulting data can be explored in a biologically relevant manner and there is flexibility to change the analysis parameters to suit different datasets and different questions.

As an example, we defined the core genes from among the coding loci present at full sequence length so that complete gene sequence information was included and p-distances could be reliably calculated. The model is designed to allow for the inclusion of genes present in <100% of genomes, which adjusts for arbitrary contig assembly issues. It is important to note that by excluding partial genes, many of which will be incomplete due to breaks in gene sequences based on sequencing technicalities and/or the genome assembly, the core genome estimates generated using our model are a conservative estimate for each bacterial species. However, as we demonstrated, simply lowering the sequence alignment to <100% will increase the number of estimated core genes, which may be appropriate for some datasets and analyses. In other words, our unit of count was the complete gene, but if the unit of count was nucleotides or the aim was to generate a list of full plus incomplete estimated core genes, then including partial sequences would be appropriate and requires the user to simply lower the sequence identity threshold in the initial analysis.

Most importantly, the process described here is completely transparent and the assumptions are easily understood, e.g. a list of genes is exported that includes the locus name, putative product, sequence length and genome position, which allows for detailed user inspection. The initial Genome Comparator output includes information about which genes are truncated (found at the ends of contigs) in each query genome and the user should evaluate these data carefully in conjunction with an assessment of the overall quality of the genome assemblies and consider whether or not to explore partial genes as part of a separate analysis. Furthermore, if a user wished to analyse amino acid sequences as opposed to nucleotide sequences, this is possible by selecting the appropriate option at the start of the Genome Comparator analysis; however, this will also significantly increase the run time and memory requirements and will become an issue with large genome datasets. A simpler option would be to convert the aligned nucleotide sequences for genes of interest into protein sequences after the Genome Comparator run is completed.

The estimated core genome sizes we obtained using our Bayesian model were expected to be lower than previous estimates, since the number of genes common to all genomes in a dataset decreases as the number of genomes increases, and our datasets are much larger and more diverse than the great majority of those previously analysed (see Table S8 for a list of relevant references). That is not a criticism of the previous studies and the analyses of small numbers of whole genomes; it is simply an indication of what data were available at the time each study was undertaken and how much the genomics field has changed in a short span of time.

We elected to demonstrate the utility of using this Bayesian model to determine which core genes are more diverse than others, but other investigators may wish to focus on the core genes that are highly conserved in a particular dataset. It is important to note that there is no one definitive “core genome” – the estimates of core genes will vary from dataset to dataset and between different methodologies.

We chose to use a Bayesian model for calculating the core genome but a frequentist model, based on a hypothesis test, could also have been applied. The advantage to using a Bayesian model is that it allows us to formalise the decision rule by a particular choice of prior. Furthermore, the Bayesian model implemented here does not account for correlations between the genes and each gene is considered to be statistically independent of each other; however, many genes are known (or likely) to be linked and operate as part of an operon or cassette. A revision of the proposed model could assess correlations between specific genes and/or gene regions. Such modifications would be a significant computational and statistical challenge given the large volume of genome data one would potentially wish to analyse, but a correlation model could provide useful biological information from the sequence data. Moreover, we selected the median as the summary of the distribution of gene pairwise distances since the underlying distributions of p-distances are not Gaussian. A better representation of the underlying probability distributions could be achieved by considering a number of different percentiles, but for this study we restricted our analyses to just the median.

Finally, the case studies we highlighted demonstrated how the data outputs and initial analyses may be used to derive more focussed analyses on specific genes or gene regions in the genome and generate new hypotheses to test. The genome diagrams are a useful way of depicting areas of potential interest in the genome and the bacterial species we evaluated here presented a huge range of possibilities for further study, from which we elected to highlight just a few as examples.

### Conclusion

The field of bacterial genomics is advancing rapidly and it is now possible to generate enormous quantities of sequence data (albeit currently incomplete) at a low cost; therefore, it is also essential to find and develop suitable, widely accessible and inexpensive methods of processing and analysing these data in order to maximise the utility and benefits of whole genome sequence data. This model formalises the estimation of the core bacterial genome as a Bayesian decision problem and the resulting outputs reveal many areas for further exploration of the bacterial core genome.

## Materials and Methods

### Datasets selected for analyses

Complete lists of the bacterial genome data included in this study, with accession numbers and available metadata (obtained by consulting the relevant published papers or websites) are listed in Datasets S1. Publicly-available whole genome sequence data for *H. pylori* (N = 107 genomes) and *S. aureus* (N = 534 genomes) were collected in two ways: 1) raw sequence trace files from the ENA were downloaded via an in-house genome assembly pipeline, assembled using Velvet [28] and uploaded to the rMLST BIGSdb database [6], [29]; and 2) finished reference genomes for each species were downloaded from GenBank and uploaded to the rMLST BIGSdb database. Only genomes that were already published in the scientific literature were included in our analyses. By comparison to MRSA genomes, few methicillin-susceptible *S. aureus* (MSSA) genomes are currently available (27 MSSA genomes were available at the time of our analysis, 18 of which were ST398) and thus we restricted the analyses to MRSA genomes only. Approximately 1000 *S. aureus* genomes were publicly available and published at the time, and we aimed to select a diverse dataset of ∼600 genomes such that the final MRSA dataset was similar in size to the *C. jejuni* and *N. meningitidis* datasets. Many of the available *S. aureus* genomes were ST239 or ST22, thus selection proceeded as follows: i) any non-ST239/ST22 genomes were automatically included; ii) among the 456 ST239/ST22 genomes available, 167 ST239 and 186 ST22 were selected (duplicates or re-sequenced genomes were removed); and iii) any genomes that were MSSA or an unknown ST (n = 54) were subsequently removed.

The Global Historical *S. pneumoniae* dataset (N = 336 genomes) included 85 assembled genomes from our previously published study [30]; sequences for 25 published genomes [31] downloaded from the ENA and assembled using Velvet; 134 genomes downloaded from GenBank; and 92 isolates sequenced and assembled as described in Protocol S1. Raw sequence data for the 616 pneumococcal genomes comprising the comparison Massachusetts data set [19] were downloaded from the ENA, assembled and uploaded to the rMLST BIGSdb database as described above.

Data for *N. meningitidis* were collected largely as part of the Meningitis Research Foundation Meningococcus Genome Library database (MRF GL; N = 514 genomes) plus 4 additional historical isolates were included [32]. The *C. jejuni* isolates included in this study (N = 601 genomes), all of human origin, were collected at the John Radcliffe Hospital in Oxford and form part of the Oxfordshire Human Surveillance collection [7]. Sequence data for *C. jejuni* and *N. meningitidis* can be found on the PubMLST [33] and rMLST BIGSdb [34] databases.

### Sequence types (STs) and clonal complexes (CCs)

STs were assigned to genomes either by retrieving the ST information from previously published papers or by extracting the sequences corresponding to the MLST loci and looking up the ST on the MLST website (*S. pneumoniae* and *S. aureus*) [35]. STs and CCs for *N. meningitidis* and *C. jejuni* were extracted from the relevant BIGSdb databases. All other CCs were defined using goeBURST [36] and the species-specific MLST databases downloaded from the MLST website. When goeBurst could not resolve the group founder, the group was assigned to ‘CC NoneX’ where X is the ST with the lowest numerical value in the group. When a lack of closely-related STs meant that a CC could not be assigned, such genomes were named ‘SingletonX’ where X corresponds to the isolate ST. Table S1 provides a summary of the STs and CCs included in this study for each bacterial species apart from *H. pylori*. Although an MLST scheme is available for *H. pylori*, the high genetic diversity of the species means that virtually every new strain has new alleles and new STs, making the interpretation of such data difficult and thus we have not defined STs and CCs for *H. pylori*.

### Genome Comparator analyses and nucleotide sequence alignments

Genome Comparator is a component of the BIGSdb genome analysis database and software suite [7]; BIGSdb facilitates whole genome analysis based on the allelic variation of individual genes. The BIGSdb Genome Comparator tool allows whole genome sequence data for one or more genomes to be compared against an annotated reference genome. The BLASTN parameters selected used a cut-off of 70% identity over a 100% alignment with a word size of 15. Potential paralogues were removed from the analyses by identifying which of the coding loci were found in more than one copy in any query genome and excluding these sequences from any further analyses. For each coding locus in the reference genome, ClustalW [37] sequence alignments were generated for all of the query genomes containing that particular sequence. Neighbor-Net diagrams were also created by Genome Comparator as part of its standard analysis and figures were created using SplitsTree [38].

### Definition of the core genome

For each of the collections of bacterial genomes, we have chosen a reference genome, consisting of a set of 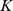 genes 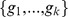. Each gene is considered independently during the analysis. Let 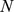 be the number of isolates under consideration. For each isolate, 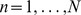, let 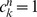 if the 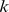-th gene is present in isolate 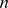 or zero if it is not present. Then, for the 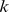-th gene, 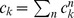 is the number of times the gene is found in 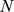 isolates. We model 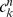 as a sequence of binomial random variables with the probability parameter 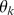. Letting 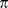 denote a probability density, the probability of observing the 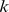-th gene 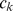 times in 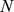 isolates given the model parameter 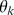 is:
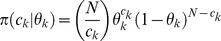
(1)The above equation, viewed as a function of 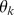, is the binomial likelihood function. We specify, for the parameter 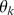, a prior probability density 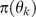. Then, using Bayes' rule, we can compute the posterior density of 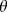 conditional on the observed frequency:
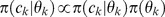
(2)If we assume the prior density 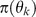 to be a beta density with parameters 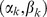 then we can combine the prior with the binomial likelihood (1) and use Bayes' rule (2) to find that the posterior density is also a beta distribution with parameters 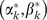 [39]. The parameters of the prior 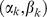 are related to the posterior parameters by 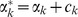 and 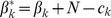.

### Decision rule

The posterior density 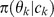 represents our uncertainty of the parameter 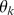. If the density 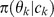 has a greater value near 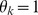 then we are inclined to believe that the gene is in the core genome. In light of this observation we introduce the following decision rule for each gene:
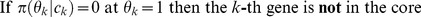
(3)The set of genes not rejected according to (3) can be defined as the estimated core genome.

### Choice of prior

The selection of a prior amounts to specifying our prior belief of whether a gene is or is not in the core genome. We might also adopt the belief that we are equally unsure of whether a gene is present in the core or not. Priors that reflect this type of belief are known as near ignorance priors [40]. Rather than selecting a near ignorance prior we argue that a prior should be selected to reflect the nature of the decision process. Prior to analysing any of the 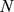 isolates we have no reason to believe that any of 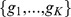 is or is not in the core. As each strain is analysed we accumulate evidence to suggest that the 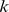-th gene is **not** a core gene. To reflect this process we adopt the prior belief that every gene is a core gene and then attempt to falsify this statement using the decision rule. Formally, this corresponds to selecting as our prior a beta density with parameters 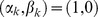.

### Calculating p-distance values and classification of estimated core genes into functional groups

Custom Perl scripts were used to split the merged sequence alignment files generated by Genome Comparator into separate sets of nucleotide sequence alignment files by estimated core gene, and then the nucleotide distance between each pair of sequences for each estimated core gene was calculated. We counted the number of sites at which the nucleotides differed between each pair of genes. We let 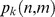 be the proportion of sites that differed between isolate 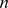 and isolate 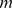 for the 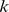-th gene in the estimated core. Then for each gene the matrix of pairwise evolutionary distances 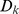 was calculated using the Jukes-Cantor model [41] where the 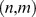 entry of the matrix 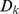 was given by:
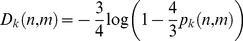
(4)The median p-distance value was then calculated as a summary statistic for each estimated core gene:
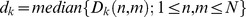
(5)For each species we collected the median distances for each gene in the estimated core. This information was plotted against the genome position of each gene (relative to the position of that locus in the reference genome) and depicted in a circular diagram created using Circos [42]. In addition, the estimated probability density function of the median pairwise evolutionary distances for each species, or for individual genes within a species, was plotted using ksdensity (Kernel smoothing function estimate) [43]. Finally, the COG functional groups of the genes with p-distance values greater than the 95^th^ percentile were determined using eggNOG [44].

### Computing requirements and open source code

The computationally intensive part of the analysis is the Genome Comparator run (because it creates sequence alignments for every gene), but this runs via a publicly-available web interface on a cluster of servers hosted at the University of Oxford. Output files are stored for one week on the server. The Bayesian model for estimating the number of core genes, the calculation of p-distance values for all reference genes, creation of the genome diagrams (Figure 3) and the generation of estimated probability graphs (Figure 4) can be implemented using freely available scripts written in the open source R software package [45]. Along with wrapper scripts that prepare the Genome Comparator outputs and a detailed manual, the relevant code is available at: https://sourceforge.net/projects/bayesianestimatedcoregenome/. As a frame of reference, the Genome Comparator analysis of the largest dataset, *C. jejuni* (601 genomes) took 90 hours to run, including all sequence alignments and generation of the Neighbor-Net diagram, but the subsequent steps took approximately 2 hours and can be run on any modestly-powered computer.

### Comparison of core genomes (*S. pneumoniae* and *S. aureus*)

For case studies 2 and 3 the names of the core genes for each of the datasets included in the comparisons were compared to each other using the VLOOKUP function in Microsoft Excel, which matches cells containing the same text (the same reference genomes were used for each dataset so the gene names were the same) in order to generate numbers of shared and unique genes. Functional groups for each of the sets of unique genes were assigned using eggNOG.

## Supporting Information

Datasets S1 Metadata for all the genomes included in this study.
(XLSX)

Figure S1 Bar graphs for each species showing gene frequency distributions.
(TIF)

Protocol S1 Description of the method for DNA extraction and whole genome sequencing of *S. pneumoniae*.
(DOCX)

Table S1 Summary of sequence types and clonal complexes for all the isolates in the study.
(XLSX)

Table S2 Comparison of estimated core genomes using different BLASTN criteria.
(DOCX)

Table S3 Potential paralogues identified for each species.
(XLSX)

Table S4 Estimated core genes and p-distances for each species.
(XLSX)

Table S5 All genes with a p-distance value greater than the 95^th^ percentile for each bacterial species and the COG functional category for each gene.
(XLSX)

Table S6 Summary of the comparison between the Bayesian and COG-based methods for the *S. pneumoniae* Massachusetts dataset.
(XLSX)

Table S7 Summary of the comparison between MRSA CC5 and CC22.
(XLSX)

Table S8 List of references for previous estimates of core genomes.
(DOCX)
