# Supplementary figures and images for: Defining the Estimated Core Genome of Bacterial Populations Using a Bayesian Decision Model
Source: PLoS Comput Biol. 2014 Aug 21;10(8):e1003788. doi: 10.1371/journal.pcbi.1003788 (PMC4140633; doi:10.1371/journal.pcbi.1003788)

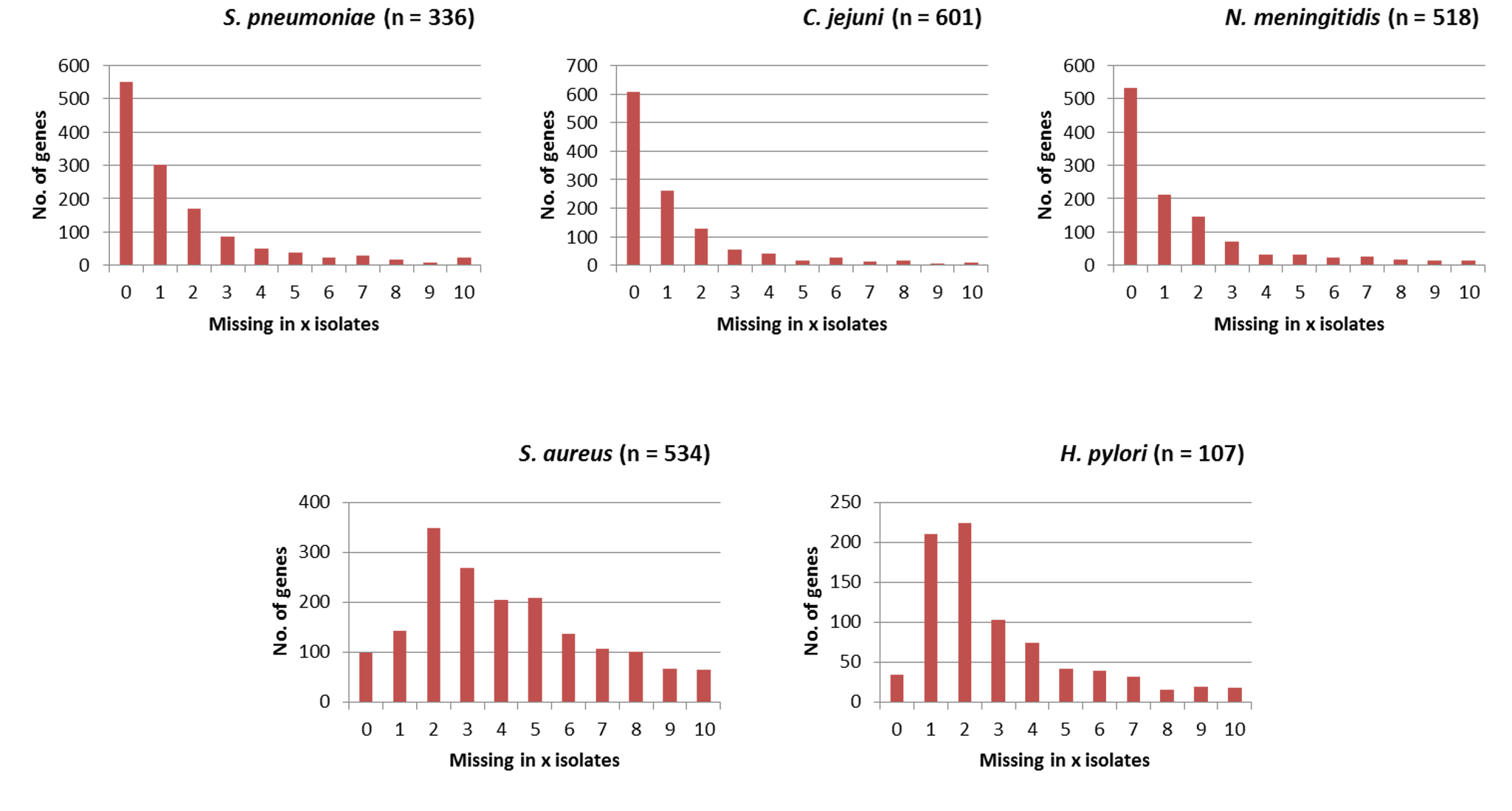

Supplement: Figure S1 — Bar graphs for each species showing gene frequency distributions. (TIF) [file pcbi.1003788.s002.tif]
