# Supplementary material for: Defining the Estimated Core Genome of Bacterial Populations Using a Bayesian Decision Model
Source: PLoS Comput Biol. 2014 Aug 21;10(8):e1003788. doi: 10.1371/journal.pcbi.1003788 (PMC4140633; doi:10.1371/journal.pcbi.1003788)
Supplement: Protocol S1 — Description of the method for DNA extraction and whole genome sequencing of S. pneumoniae. (DOCX) [file pcbi.1003788.s003.docx]

**DNA extraction and whole genome sequencing of *S. pneumoniae* genomes**

Single pneumococcal colonies were cultured on Columbia agar with sheep blood (Oxoid) and incubated overnight at 37ºC plus 5% CO_2_. The resulting growth was then subcultured onto tryptic soy agar (Oxoid) with 1000 U/ml catalase (Sigma-Aldrich) before further incubation overnight at 37ºC plus 5% CO_2_. Pneumococcal growth was then suspended in 1ml phosphate buffered saline (pH 7.4, Fisher Scientific) and centrifuged at 7600 rpm for ten minutes. A Qiagen DNeasy Blood & Tissue Kit was then used to complete the DNA extractions, following the manufacturer’s instructions. A final sample of at least 20 ng/µl extracted DNA was obtained for each of 95 pneumococcal isolates and sent for sequencing at the Wellcome Trust Sanger Institute. Standard Illumina multiplex library construction with a 200 bp insert size followed by 100 bp paired-end sequencing on the Illumina HiSeq platform were performed. Sequence reads were assembled using Velvet; assemblies for three isolates were of poor quality and were removed from further analyses.
