# Supplementary material for: Defining the Estimated Core Genome of Bacterial Populations Using a Bayesian Decision Model
Source: PLoS Comput Biol. 2014 Aug 21;10(8):e1003788. doi: 10.1371/journal.pcbi.1003788 (PMC4140633; doi:10.1371/journal.pcbi.1003788)
Supplement: Table S2 — Comparison of estimated core genomes using different BLASTN criteria. (DOCX) [file pcbi.1003788.s005.docx]

Table S2. Comparison of estimated core genomes using different BLASTN alignment criteria.

|  |  |  | 70% identity / 90% alignment | | | | 70% identity / 100% alignment | | | |
| --- | --- | --- | --- | --- | --- | --- | --- | --- | --- | --- |
| Bacterial  species | Reference genome | Reference genome size (Mb) | Unique genes (n) | Potential paralogues in whole genome (n) | % of genomes that possess each core gene | Estimated core genes (n) | Unique genes (n) | Potential paralogues in whole genome (n) | % of genomes that possess each core gene | Estimated core genes (n) |
|  |  |  |  |  |  |  |  |  |  |  |
|  |  |  |  |  |  |  |  |  |  |  |
| *S. pneumoniae* | ATCC700669 | 2.22 | 3 | 60 | ≥99.7 | 1113 | 6 | 40 | ≥99.7 | 851 |
| *C. jejuni* | NCTC11168 | 1.64 | 3 | 6 | ≥99.8 | 1183 | 6 | 1 | ≥99.8 | 866 |
| *N. meningitidis* | FAM18 | 2.19 | 4 | 50 | ≥99.8 | 1104 | 7 | 21 | ≥99.8 | 744 |
| *S. aureus* | HO5096_0412 | 2.83 | 1 | 38 | ≥99.8 | 537 | 4 | 13 | ≥99.8 | 242 |
| *H. pylori* | 26695 | 1.67 | 21 | 15 | ≥99.1 | 766 | 45 | 2 | ≥99.1 | 244 |
|  |  |  |  |  |  |  |  |  |  |  |
