# Supplementary material for: Defining the Estimated Core Genome of Bacterial Populations Using a Bayesian Decision Model
Source: PLoS Comput Biol. 2014 Aug 21;10(8):e1003788. doi: 10.1371/journal.pcbi.1003788 (PMC4140633; doi:10.1371/journal.pcbi.1003788)
Supplement: Table S8 — List of references for previous estimates of core genomes. (DOCX) [file pcbi.1003788.s011.docx]

| **Table S8.** List of references to previously published papers that estimated a bacterial core genome. | | | | | | |
| --- | --- | --- | --- | --- | --- | --- |
|  |  |  |  |  |  |  |
| Species | First author | Year | PubMed  ID no. | No. of estimated  core genes | No. of whole genomes | Comments |
| *S. pneumoniae* | Donati | 2010 | 21034474 | 1666 | 44 |  |
|  | Hiller | 2011 | 22205975 | 1324 | 52 |  |
|  | Croucher | 2013 | 23644493 | 1194 | 616 |  |
|  |  |  |  |  |  |  |
| *C. jejuni* | Lefubure | 2010 | 20688752 | ~1300 | 43 |  |
|  | Friis | 2010 | 20865039 | 1295 | 13 |  |
|  | Biggs | 2011 | 22096527 | 1001 | 2 |  |
|  |  |  |  |  |  |  |
| *N. meningitidis* | Hotopp | 2006 | 17159225 | 1706 | 3 |  |
|  | Schoen | 2008 | 18305155 | 1337 | 6 |  |
|  | Schoen | 2009 | 19477564 | 1330 | 7 |  |
|  | Rusniok | 2009 | 19818133 | 1736 | 5 |  |
|  | Budroni | 2011 | 21368196 | 1630 | 20 |  |
|  | Krauland | 2012 | 22558202 | 1776 | 2 |  |
|  | Kong | 2013 | 23902748 | 1090 | 36 |  |
|  |  |  |  |  |  |  |
| *S. aureus* | Hall | 2010 | 20019077 | 1923 | 14 |  |
|  | Harris | 2010 | 20093474 | ??? | 63 | ST239 only |
|  | Castillo-Ramirez | 2011 | 21779170 | 1492 | 63 | ST239 only, follow on from Harris study above |
|  | Boissy | 2011 | 21489287 | 2266 | 17 |  |
|  | Castillo-Ramirez | 2012 | 23270620 | ??? | 165 | ST239 only; includes 62 from Harris study above |
|  | Holden | 2013 | 23299977 | ??? | 193 | ST22 only |
|  |  |  |  |  |  |  |
| *H. pylori* | McClain | 2009 | 19123947 | 1237 | 5 |  |
|  | Fischer | 2010 | 20478826 | 1223 | 7 |  |
|  | Kawai | 2011 | 21575176 | 1079 | 20 |  |
|  | Lara-Ramirez | 2011 | 21387011 | 1186 | 9 |  |
|  | Gunaletchumy | 2012 | 23012278 | 760 | 10 |  |
